# Laminopathy-causing lamin A mutations reconfigure lamina-associated domains and local spatial chromatin conformation

**DOI:** 10.1080/19491034.2018.1449498

**Published:** 2018-03-08

**Authors:** Nolwenn Briand, Philippe Collas

**Affiliations:** a Department of Molecular Medicine, Institute of Basic Medical Sciences, Faculty of Medicine, University of Oslo, Oslo, Norway; b Norwegian Center for Stem Cell Research, Department of Immunology and Transfusion Medicine, Oslo University Hospital, Oslo, Norway

**Keywords:** chromatin, differentiation, LAD, lamin A/C, genome conformation, 3D genome

## Abstract

The nuclear lamina contributes to the regulation of gene expression and to chromatin organization. Mutations in A-type nuclear lamins cause laminopathies, some of which are associated with a loss of heterochromatin at the nuclear periphery. Until recently however, little if any information has been provided on where and how lamin A interacts with the genome and on how disease-causing lamin A mutations may rearrange genome conformation. Here, we review aspects of nuclear lamin association with the genome. We highlight recent evidence of reorganization of lamin A-chromatin interactions in cellular models of laminopathies, and implications on the 3-dimensional rearrangement of chromatin in these models, including patient cells. We discuss how a hot-spot lipodystrophic lamin A mutation alters chromatin conformation and epigenetic patterns at an anti-adipogenic locus, and conclude with remarks on links between lamin A, Polycomb and the pathophysiology of laminopathies. The recent findings presented here collectively argue towards a deregulation of large-scale and local spatial genome organization by a subset of lamin A mutations causing laminopathies.

## Introduction

The nuclear genome is enclosed and protected by the nuclear envelope, a double-membrane system perforated by nuclear pores and underlined by the nuclear lamina, a meshwork of intermediate filaments called nuclear lamins [1]. Evidence accumulated over three decades indicates that both A- and B-type lamins play important roles in the regulation of DNA replication, gene expression and 3-dimensional (3D) organization of the genome. Lamins B1 and B2 are localized at the peripheral nuclear lamina, where they interact with extended chromatin domains called lamina-associated domains (LADs) [2,3]. Lamins A and C (also called lamin A/C because both are splice variants of the *LMNA* gene, and abbreviated here as lamin A) are found both at the nuclear periphery and in the nuclear interior where they exist as a detergent-soluble pool [4]. Lamin A is also able to interact with chromatin both at the nuclear periphery and in the nuclear interior [5–7]. In doing so, lamin A has been shown to contribute to the regulation of progenitor cell differentiation [8]. In this review, we discuss patterns of lamin interactions with the genome and how these may modulate developmental gene expression.

Nuclear lamins are linked to disease. In the course of nearly two decades, over 400 mutations throughout the *LMNA* gene have been linked to various forms of laminopathies, including muscle dystrophies [9] and partial lipodystrophies [10–13]. The heterozygous lamin A p.Arg482Trp (R482W) point mutation is considered to be a hot-spot mutation causing familial partial lipodystrophy of Dunnigan type (FPLD2) [14]. FPLD2 is characterized by adipose tissue atrophy in the lower body, upper body fat accumulation, muscle hypertrophy and metabolic disorders including glucose intolerance and insulin insensitivity leading to the metabolic syndrome and type-2 diabetes mellitus [14,15]. FPLD2 patients also present severe atherosclerosis leading to cardiovascular events [16–18]. Investigations in mice and in various cellular models of the disease agree in that the lamin A mutation leads to defective adipose differentiation [19–22].

Mechanisms by which the lamin A R482W or other mutations (e.g. R482Q/L to name just these) give rise to the FPLD2 phenotype remain largely unknown. Laminopathy-causing mutations have been shown to involve defects in nuclear and cellular mechano-sensitivity [23], defective signal transduction pathways [24], mis-sequestration of transcription factors at the nuclear envelope [21] and alterations in global nuclear architecture [25]. The latter likely originates from deformations of the nuclear envelope and as discussed below, altered interactions of nuclear lamins with chromatin [26].

Here, we review general aspects of nuclear lamin association with the genome, with an emphasis on lamin A-chromatin interactions. We address recent evidence of large-scale reorganization of lamin A LADs in cells expressing lamin A mutations and implications thereof on spatial rearrangement of chromatin in cellular models of FPLD2. We discuss how the hot-spot lipodystrophic lamin A R482W substitution alters epigenetic states and chromatin conformation at an anti-adipogenic microRNA locus in adipose progenitor cells. We conclude with notes on connections between lamin A, Polycomb regulation of gene expression and the pathophysiology of laminopathies. Observations outlined here point to a deregulation of spatial chromatin organization on a global scale and at the gene level by a subset of lamin A mutations causing laminopathies.

### Patterns of nuclear lamin association with the genome

**Figure 1.**
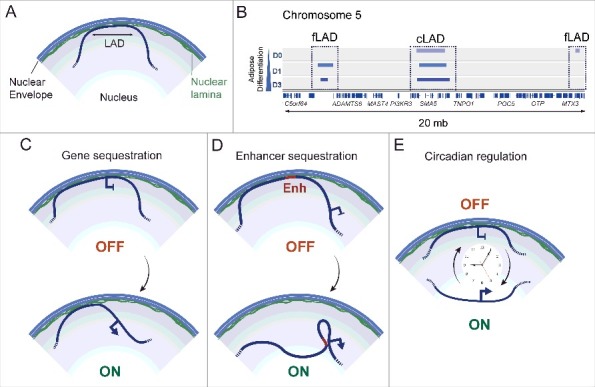
Modes of regulation of gene expression by nuclear lamins. (A) A lamina-associated domain (LAD). (B) Genome browser view of lamin A LADs during adipose differentiation of human adipocyte progenitors into adipocytes (day 0, 1, 3 of differentiation). Facultative fLADs and constitutive cLADs are shown in a region of chromosome 5. LAD data are from reference [31]. (C-E) Regulation of developmental gene expression by (C) sequestration/release of a whole gene locus at/from the nuclear envelope, (D) sequestration/release of an enhancer at/from the nuclear envelope and (E) circadian association a gene locus with, and release from, the nuclear envelope.

LADs represent defined regions of the genome that associate with A- or B-type lamins predominantly at the nuclear envelope (Fig. 1A). Lamin B1 LADs have first been identified by expression of lamin B1 fused with the bacterial Dam methylase as a means of eliciting adenine methylation in DNA in close proximity to lamin B1 [27]. This approach (DamID) has identified LADs as 0.1 to 100 kilobase (kb) regions that are gene-poor, AT-rich and enriched in histone H3 dimethylated on lysine 9 (H3K9me2), a mark of heterochromatin [27]. Hence, genes found in LADs are repressed or expressed at a much lower level than genes outside LADs. LADs with similar properties have also been identified by chromatin immunoprecipitation-sequencing (ChIP-seq) of lamin B1 and lamin A/C in various cell types [7,28–31].

On a technical note, nuclear lamins are notoriously difficult to immunoprecipitate, and their enrichment level in chromatin detected by ChIP is usually low. Lamins also display broad genome occupancy profiles, in contrast to transcription factors or most histone modifications. Therefore, the most commonly used peak calling algorithms will not successfully detect LADs; this requires a well-suited domain (rather than peak) detection algorithm which discriminates broad low-level enrichment from noise [30].

The localization of B-type lamins at the nuclear lamina predicts that lamin B1 LADs represent chromatin domains anchored at the nuclear periphery. A paradox arises then, considering that LADs make up 25–30% of the genome [27,30,32]. Yet, there is arguably no sufficient ‘space’ to physically juxtapose nearly a third of the genome to the nuclear lamina in a given cell. Resolving this issue, single-cell genome-wide mapping of lamin B LADs [33], supplemented by fluorescence in situ hybridization visualization of a pool of LADs [34] reveals that 30% of LADs detected by sequencing approaches localize at the nuclear periphery in single cells [34]. Thus, LADs represent parts of the genome that are not all necessarily anchored at the nuclear lamina simultaneously in all cells in a population. Rather, LADs appear to be regions that dynamically bind to and dissociate from the nuclear lamina, and which and after cell division do not always mirror all LADs in previous cell generations [34].

Patterns of lamin A association with the genome are arguably more challenging to interpret than for lamin B because A-type lamins also exist in the nuclear interior. Accordingly, a ChIP-seq analysis of A- and B-type lamins in HeLa cells highlights overlaps and differences in genome regions cross-linkable to lamins A and B [6]. A significant proportion of lamin A/C LADs (hereafter, lamin A LADs) include unique gene-rich and transcriptionally active euchromatic regions, unlike lamin B LADs which are typically heterochromatic [6]. Similar features of lamin A LADs have been reported in mouse cells [7]. These findings suggest that lamin A-chromatin interactions may serve a distinct regulatory role on gene expression when compared to lamin B LADs.

### Developmental regulation of lamin-chromatin interactions

Comparison of LADs between cell types and between species strikingly reveals that LADs constitute overall well-conserved regions of the genome [32]. These constitutive LADs (cLADs) differ from the more variable, or facultative, fLADs which tend to be smaller, more gene-rich and vary between cell types. Constitutive and facultative LADs have been identified by lamin B1 DamID in differentiating mouse embryonic stem cells [35] and by ChIP-seq of lamin A/C in human adipocyte progenitor cells induced to differentiate into adipocytes [31] (Fig. 1B). Since LADs constitute an overall transcriptionally repressive environment, dynamic lamin-chromatin associations may constitute a mechanism regulating differentiation-coupled changes in gene expression.

Several recent lines of evidence promote a view of nuclear envelope proteins regulating developmental gene expression in a cell type-specific manner. For instance, repositioning of genes to, or away from, the nuclear periphery has elegantly been demonstrated to constitute a spatial mechanism regulating myogenic gene expression in mouse myoblasts [36]. Release of neuronal genes from the nuclear lamina during neuronal differentiation of embryonic stem cells [35], or detachment of adipogenic control genes from lamin A after adipogenic induction of adipocyte progenitors [5,31], further support the idea of release, or ‘unlocking’, of developmental loci from the nuclear lamina as a pre-requisite for transcriptional activation [2,35,37] (Fig. 1C). Differential lamina-chromatin interactions have also been implicated in the regulation of expression of immune function-related genes in T cells [38]. This level of regulation strikingly involves a sequestering of enhancers at the nuclear lamina, away from genes they are meant to activate (Fig. 1D). Induction of these genes upon T cell activation coincides with the release of enhancers from the nuclear periphery, enabling contacts with their cognate promoters [38]. These findings raise an attractive model of gene expression regulation at the nuclear periphery by reversible sequestration of regulatory elements at the nuclear envelope as a means of keeping genes turned off.

### Metabolic connection: Circadian lamin-chromatin associations

Not only associations of genes with the nuclear lamina can be developmentally regulated, they also exhibit oscillating patterns regulating expression of circadian genes [39]. Cell-autonomous clocks generate intrinsic 24-h oscillations in gene and protein expression patterns that control many time-dependent signaling and metabolic processes [40]. Interestingly, a subset of circadian genes is recruited to the nuclear lamina in a circadian manner – that is, their localization at the nuclear periphery oscillates with a ∼24-h period, in a process driven by poly [ADP-ribose] polymerase 1 (PARP1) and CCCTC-binding factor (CTCF) [39]. Whether entire LADs, as opposed to isolated genes, also display circadian rhythmicity is currently unknown, but the recent findings indicate that at least punctual interactions of genes with the nuclear lamina mediate ‘circadian transcriptional plasticity’ [39] (Fig. 1E). Because circadian rhythms are intimately linked to cell metabolism [40], they therefore connect cellular metabolic state to the regulation of lamin-genome interactions. This raises the hypothesis that metabolic alterations in laminopathy patients [14,15] could derail lamin-genome interactions, and thereby gene expression networks, in patients cells and in cellular models of laminopathies. To what extent laminopathy-causing lamin A mutations affect circadian rhythms in affected tissues is a promising area of investigation which may shed new light on the pathophysiology of these diseases.

### Lamin A mutations causing laminopathies reorganize lamin A-chromatin interactions

Laminopathies affect specific tissues by mechanisms that remain largely unknown [41]. Lamin A plays a fundamental role in the distribution of euchromatin and heterochromatin [42], in the restriction of chromatin mobility [43] and in developmental gene expression [44–46]. Lamin A also exhibits cell type-specific differentiation-dependent interactions with chromatin [31]. This raises the possibility that at least some lamin A mutations alter interactions with chromatin in distinct parts of the genome and in a cell type-specific manner.

**Figure 2.**
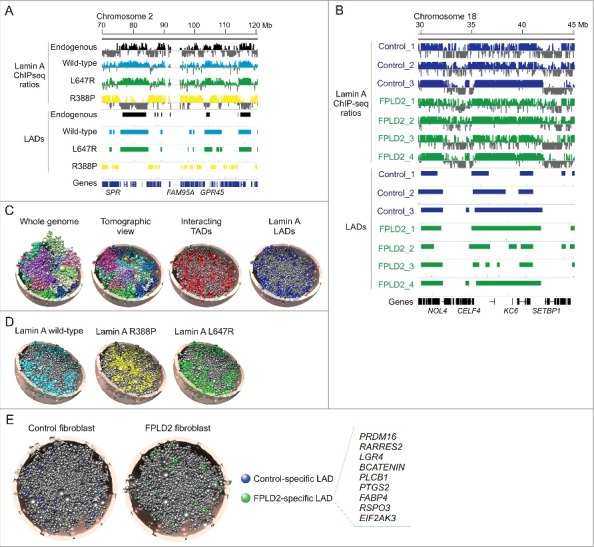
3D genome modeling provides a spatial appreciation of LADs altered by lamin A mutations causing laminopathies. (A) Genome browser view of lamin A ChIP-seq profiles and LADs in a region of chromosome 2 in HeLa cells expressing indicated lamins. (B) Lamin A ChIP-seq profiles and LADs in a region of chromosome 18, in three control and four FPLD2 patient fibroblast cultures, the latter all with the R482W mutation. LAD data in (A) and (B) are from reference [26]. (C) A 3D structural model of HeLa cell nuclei. The whole-genome model reflects chromosome territories; each chromosome is modeled as a chain of beads representing TADs, and is colored differently. Chromosome territories are also visible in the tomographic view. Tomographic views of the same genome structure also reveal TADs interacting pairwise (red) and TADs containing LADs (blue) (adapted from reference [26). (D) 3D genome models showing LADs generated by expression of indicated lamins in HeLa cells [26]. (E) 3D genome models showing LADs specific to control and FPLD2 fibroblasts with the lamin A R482W mutation (from reference [26). Box, genes pertaining to white and brown adipogenesis found in FLPD2-specific LADs. Nucleus radius in the models in (C-E) is 5 µm. All panels were from reference [26] and were used with permission.

A first indication that lamin A mutations can alter global chromatin organization comes from the mapping of lamin A LADs associated with expression of wild-type and mutated lamin A in cellular models of laminopathies. Overexpression of Flag-tagged versions of wild-type lamin A, a lamin A mutant causing a progeroid disorder (L647R) [47], and a lamin A mutant linked to congenital muscle dystrophy and lipodystrophy (R388P) [48], results in distinct lamin A-genome interactions detectable by ChIP-seq of the Flag tag [26]. Whereas LADs linked to wild-type lamin A and the L647R mutant broadly overlap and display typical gene-poor LAD characteristics, they show little overlap with R388P LADs (Fig. 2A). The latter are in contrast gene-rich, smaller and mainly found in euchromatin and active parts of the genome. These results suggest that laminopathy-causing mutations can significantly alter the landscape of lamin-chromatin associations.

Expression of wild-type lamin A, lamin A R482W causing FPLD2 and lamin A R453W causing Emery-Dreifuss muscle dystrophy in myoblasts was intriguingly shown to result in up to over 2,500 differential LADs between these three constructs [49]. Several factors may explain this unexpected number, including overexpression of the lamins as Dam fusion proteins and the use of a peak calling algorithm not particularly well suited for LADs [49]. Balancing this study, differences in lamin A LADs reported by ChIP-seq of normal and FPLD2 patient fibroblasts with the lamin A R482W mutation are minor [26] (Fig. 2B). So to what extent overexpression studies [26,49] reflect disease situations remains to be examined more closely. It is also at present unclear how the lamin A R453W mutation would cause a rearrangement of LADs [49], but this could be due to structural defects in the nuclear lamina or ectopic binding to chromatin.

Collectively, these studies provide evidence that expression of disease-causing lamin A mutations can drastically alter interactions with chromatin. As discussed below, evidence for disease-specific LADs suggests a spatial reorganization of the genome by alterations of the radial (i.e. center vs. peripheral) positioning of chromatin domains.

### Three-dimensional genome modeling enables predictions of radial positioning of LADs

Whereas a linear (1D) examination of LADs (Fig. 2A) provides insights on the distribution of LADs along the genome, it lacks information on where LADs are localized in the 3D nucleus space. Advances in genome-wide mapping of chromosome-chromosome interactions and in methods to model whole genomes in 3D enable predictions of where LADs might be found in 3D space, and on the impact of lamin mutations on spatial genome organization.

Coupling high-throughput sequencing with chromosome conformation capture assays such as Hi-C [50,51] makes it possible to map genomic interactions within chromosomes and between chromosomes in entire genomes. These methods are based on crosslinking proximal DNA molecules, ligating them together and sequencing the interacting DNA fragments [50]. The output from these experiments is a matrix of pairwise chromosomal contact frequencies from which a 3-dimensional representation of the genome can be inferred [51].

From the analysis of Hi-C data, a hierarchical organization of the eukaryotic genome has emerged. The genome can be broadly categorized into open, or active, ‘A’ compartments, and closed, or repressed, ‘B’ compartments [52]. Within compartments, chromatin is organized into topologically-associated domains, or TADs [53,54]. TADs are defined as regions with a high frequency of chromosomal contacts, whereas contacts are less frequent between adjacent TADs. TAD boundaries are overall well conserved along the linear genome between cell types [52] and during differentiation [55,56], suggesting that TADs constitute structural entities of the genome. TADs also partially specify regulatory interactions between promoters and enhancers [53,54] and thus may act as gene regulation units.

A strategy to investigate 3D genome conformation is to computationally model 3D structures of the genome and analyze the properties of these structures. 3D genome modeling has been applied at various scales (from single loci to whole genomes) and resolutions (from megabases down to tens of kilobases) [57–60]. One approach to modeling genomes from Hi-C data is to reconstruct a single consensus 3D genome model that represents an average of all structures in the cell population under study [61–63]. Other modeling methods recapitulate variations in genome conformation across a population of cells by simulating hundreds or thousands of genome structures [57–59]. Recent 3D genome modeling frameworks are also designed to incorporate TAD positional constraints, in addition to chromosomal contacts, such as TAD interactions with nuclear lamins [26,64]. Such LAD data provide radial information for the placement of genomic domains during the modeling process. It should be noted however that LADs are not necessarily strictly localized at the nuclear periphery (notably due to the nucleoplasmic pool of A-type lamins and to LADs being ascribed to the periphery of nucleoli [65]. Thus in 3D genome modeling, some LADs may be ascribed to the nuclear interior, towards to the edge of nucleoli. This property could conceivably be taken into account as a positional constraint for TADs or other genomic domains in 3D genome modeling exercises.

Interestingly, a recent genome architecture mapping (GAM) approach has been reported, which physically measures chromatin contacts and other properties of 3D chromatin conformation in a large number of thin sections across nuclei [66]. These measurements are used to reconstruct 3D contact matrices. Remarkably, GAM and Hi-C contact matrices strongly correlate [66], suggesting that 3D genome models could also be derived from GAM datasets.

We have recently introduced Chrom3D, a genome 3D modeling platform that integrates positional constraints for TADs based on their interaction with, for example, nuclear lamins [26] (Fig. 2C). Combining Hi-C and LAD data enables quantitative estimations of the genome-wide radial positioning of TADs in 3D structures. Several features of spatial genome organization emerge from analyses of such models. For example, the models respect the notion of chromosome territories stemming from imaging analysis [67], even though this was not entered as a constraint in the modeling (Fig. 2C, left). Further, large and relatively gene-poor (and AT-rich) chromosomes are more stably positioned towards the nuclear periphery than smaller more gene-rich (and GC-rich) chromosomes. Importantly, analysis of 3D models also quantitatively recapitulates the radial placement of LADs identified in single cells [33,34] (Fig. 2C, right). Equipped with 3D genome modeling techniques, we are now able to visualize and predict the spatial distribution of defined genomic domains or genes, and investigate mechanisms of spatial gene regulation in diseases susceptible to affect spatial chromatin organization, such as laminopathies.

### Spatial attribution of LADs

Applying 3D genome modeling to Hela cell models of laminopathies reveals striking differences in the spatial distribution of LADs linked to various lamin A mutations [26]. There, Chrom3D was applied from HeLa cell high-resolution Hi-C data [52] and lamin A ChIP-seq dataset generated in our laboratory after overexpression of wild-type or mutated lamin A in HeLa cells. Incorporation of wild-type and mutant lamin A LAD information into Chrom3D models of HeLa nuclei shows that lamin A R388P LADs map more frequently to the nuclear center than wild-type or L647R lamin A LADs [26], as exemplified in Fig. 2D. Immunofluorescence labeling of the lamin mutants could arguably predict a differential LAD distribution [26], but this method provides no indication on chromatin association. The high gene content and smaller size of R388P LADs than wild-type or L467R LADs agree with the radial positioning of these LADs inferred from Chrom3D models [26].

More directly relevant for laminopathies, 3D modeling of fibroblasts from FLPD2 patients also reveals unexpected features compared to normal fibroblasts [26]. There again, 3D models were generated from published Hi-C data for IMR90 fibroblasts (as a proxy for skin fibroblasts) [52] and lamin A LADs were mapped by us by ChIP-seq of lamin A in patient and control fibroblasts [26]. The linear distribution of FPLD2 lamin A R482W LADs is overall similar to that of control LADs (Fig. 2B). However, analysis of 3D models shows that FPLD2-specific LADs (LADs unique to FPLD2 fibroblasts) are positioned more centrally than all LADs in these cells (Fig. 2E). This patient-specific lamin A-chromatin association is nevertheless accompanied by a partial yet significant repositioning of these LADs towards (but not *at*) the nuclear periphery. Conversely, the loss of LADs is linked to a repositioning of these regions away from the periphery. A view arising from 3D genome modeling therefore implies an unexpected deregulation of lamin-genome interactions in the nuclear interior and not necessarily at the nuclear envelope, by laminopathy-causing lamin A mutations.

The functional significance of differential LAD positioning for laminopathies remains to be investigated. LADs gained or lost specifically in FPLD2 patient fibroblasts notably contain genes pertaining to white and brown adipocyte differentiation [26] (Fig. 2E), and could perhaps relate to the adipogenic and metabolic phenotypes of patients [15]. Ectopic lamin A binding to promoters [5] could also perturb transcriptional regulation in patient tissues. With the emergence of increasingly performant 3D genome modeling platforms, we promote the view that introducing a spatial component in the study of the genomics of laminopathies will lead to a deeper understanding of the impact of lamin mutations on gene regulation in cell type-specific contexts.

### The lipodystrophic lamin A R482W mutation alters 3D genome conformation

In line with a role of lamin A in the determination of progenitor cells fate [8,20,46,68], expression lamin A R482W in human adipocyte progenitors not only impairs adipogenic differentiation but also rewires gene expression towards a myogenic phenotype [20]. Previous studies from our laboratory point to multiple levels of lamin A-dependent regulation of adipogenesis. Induction of differentiation induces a redistribution of LADs at the nuclear periphery [31] and a punctuate association or dissociation of promoters from lamin A [5], coordinating an adipogenic gene expression program.

Whereas the R482W substitution does not severely disrupt the structure of the immunoglobulin-like fold and oligomerization properties of the lamin, it affects its interaction with DNA [69] and with protein partners [21]. Interestingly, the mutation does not elicit a major disruption of peripheral LADs in FPLD2 patient cells, but affects LADs in the nuclear interior [26]. Thus, defective adipogenesis in FPLD2 could result from deregulation of gene expression involving the nucleoplasmic pool of mutated lamin A. Indeed, impaired differentiation of adipocyte progenitors harboring lamin A R482W concurs with a lack of repression of the anti-adipogenic *MIR335* gene (encoding the micro-RNA miR-335) in the nuclear interior [22]. Specific inhibition of miR-335 rescues differentiation in mutant cells, suggesting that miR-335 overexpression may be relevant in the pathophysiology of FPLD2.

Deficiencies in *MIR335* gene repression by lamin A R482W appear at several levels [22]. (i) Intranuclear positioning. Adipogenic induction repositions the *MIR335* locus towards the nuclear periphery in cells expressing wild-type lamin A; this concurs with lamin A binding to the locus and *MIR335* downregulation. In contrast, the R482W mutation abolishes lamin A binding to the locus and *MIR335* repositioning. This suggests a role of nucleoplasmic lamin A in the determination of locus positioning in space and impairment of this function by the mutation.

(ii) Promoter-enhancer interactions. Lamin A-chromatin interactions modulate local chromatin conformation: whereas expression of wild-type lamin A correlates with low frequency overlap of the *MIR335* gene with *MIR335* enhancers (as shown by fluorescence in situ hybridization), the R482W mutation elicits a high-frequency of enhancer-gene overlap concordant with over-expression of *MIR335*. This suggests that the lamin A mutant causes a conformational remodeling of the locus favoring its over-expression. The rigidity of chromatin provided by an intranuclear lamin A scaffold [43] possibly contributes to restricting local chromatin looping events.

(iii) Epigenetic deregulation. Defective lamin A R482W binding to chromatin is also linked to epigenetic alterations of the *MIR335* promoter and regulatory elements (enhancers) after adipogenic induction [22]. These include decreased H3K27me3 deposition and increased H3K27 acetylation, again in line with *MIR335* overexpression. Decreased H3K27me3 in lamin A mutant cells suggests a defective Polycomb repressor complex 2 (PRC2) recruitment or stabilization by the lamin A R482W mutant at the *MIR335* locus.

These findings indicate that lamin A is implicated in the developmental regulation of (anti)-adipogenic gene expression by promoting (i) relocalization of loci towards the repressive nuclear periphery, (ii) restricting promoter-enhancer interactions, and (iii) scaffolding epigenetic modifying complexes at relevant loci. These genome-related functions of lamin A are consistent with a rewiring of chromatin loops (promoter-enhancer contacts) inside TADs upon adipose differentiation [55]. The recent findings argue that lamin A mutants are able to affect lineage-specific loci in addition to more global structural changes in the genome.

## Conclusions and perspectives

### Lamin mutations, Polycomb and pathophysiology of lipodystrophic laminopathies

**Figure 3.**
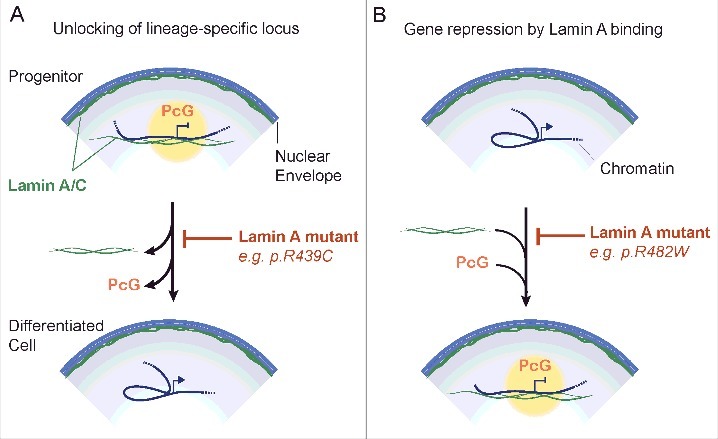
Connecting lamin A mutations, Polycomb and local genome conformation to developmental gene expression. (A) Unlocking of a lineage-specific gene from lamin A. In progenitor cells, a locus is held in a repressed state by Polycomb proteins (PcG) stabilized intranuclear lamin A/C. Dissociation of lamin A/C from the locus upon differentiation favors release of Polycomb, promoter-enhancer interaction and transcriptional activation of the locus. Some lamin A mutants such as lamin A p.R439C [72] enhance lamin A binding to chromatin and would inhibit this process. (B) Conversely, a gene active in progenitor cells is repressed by Polycomb on differentiation. This is enabled by an intranuclear lamin A network which stabilizes Polycomb at the locus. An example is the *MIR335* gene in adipocyte progenitors [22]. The lamin A p.R482W mutant prevents lamin A binding, Polycomb recruitment and transcriptional repression.

Data support a role of lamin A in targeting PRC2 complexes to specific loci in mesenchymal progenitor cells [70]. A fraction of PRC2 interacts with lamin A [71], suggesting a view of nucleoplasmic lamin A providing a scaffold for recruitment and/or stabilization of Polycomb at target loci in the nuclear interior during differentiation (Fig. 3). A plausible scenario is that Polycomb recruitment to target genes is affected by lamin A mutations which either enhance (lamin A p.R439C) [72] (Fig. 3A) or decrease (lamin A p.R482W) (Fig. 3B) lamin association with chromatin. Down-regulation of lamin A in myoblasts redistributes PRC2 complexes and leads to ectopic expression of Polycomb targets and premature activation of myogenic differentiation [70]. Considering how lamins and the nuclear envelope participate in regulating adipogenic and myogenic gene expression [20,22,36,45], we speculate that tissue-specific genomic localization of lamin A-PRC2 interactions may be mediated by as of yet unidentified tissue-specific factors.

Defective PRC2 association with specific loci might provide a unifying mechanism for the pathophysiology of laminopathies. Several lines of evidence support this hypothesis. (i) Both EZH2 (the histone H3K27 methylase of the PRC2 complex) and H3K27me3 levels are globally reduced in cells from Hutchinson-Gilford Progeria Syndrome patients [73]. (ii) PRC2 regulates expression of the tumor suppressors *P16/INK4A* and *P21* [74], potentially linking laminopathies to premature senescence, a hallmark of the disease [15]. (iii) PCR2 also modulates mesenchymal lineage specification by repressing *WNT* genes [75] through the H3K27 methylase activity of EZH2 [76,77]. In doing so, PRC2 directly represses the anti-adipogenic Wnt/β-catenin pathway, promoting adipogenesis over other cell fates [78]. (iv) EZH2 regulates skeletal muscle differentiation [79], which requires a proper lamin A network [70]. We propose from these studies that PRC2 mis-localization elicited by lamin A mutations affects the balance between proliferation and lineage-specific differentiation in tissue-specific progenitors, leading to tissue-specific phenotypes.

The combination of predictive 3D genomics, imaging approaches and functional studies in early (e.g. pluripotent cell-derived) and late (tissue-specific progenitor cell-derived) developmental models of laminopathies is likely to pave the way to an unprecedented understanding of these diseases and to the development of potential therapeutics.

## Abbreviations

ChIP-seq: chromatin immunoprecipitation sequencing
FPLD2: familial partial lipodystrophy of Dunnigan-type
LAD: lamina-associated domain
TAD: topologically-associated domain
